# Overlooked FANCD2 variant encodes a promising, portent tumor suppressor, and alternative polyadenylation contributes to its expression

**DOI:** 10.18632/oncotarget.14989

**Published:** 2017-02-01

**Authors:** Bing Han, Yihang Shen, Piyan Zhang, Panneerselvam Jayabal, Raymond Che, Jun Zhang, Herbert Yu, Peiwen Fei

**Affiliations:** ^1^ University of Hawaii Cancer Center, University of Hawaii, Honolulu, HI, USA; ^2^ Department of Laboratory Medicine and Pathology, Mayo Clinic, Rochester, MN, USA; ^3^ Graduate Program of Molecular Biosciences and Bioengineering, University of Hawaii, Honolulu, HI, USA

**Keywords:** polyadenylation site (PAS), tumor suppressor, Fanconi anemia, FANCD2, FANCD2-V1

## Abstract

Fanconi Anemia (FA) complementation group D2 protein (FANCD2) is the center of the FA tumor suppressor pathway, which has become an important field of investigation in human aging and cancer. Here we report an overlooked central player in the FA pathway, FANCD2 variant 2 (FANCD2-V2), which appears to perform more potent tumor suppressor-function compared to the known variant of FANCD2, namely, FANCD2-V1. Detailed analysis of the FANCD2 gene structure indicated a proximal and distal polyadenylation site (PAS), associated with V2 and V1 transcripts accordingly. RNA polymerase II Chromatin immunoprecipitation (ChIP) targeting the two PAS-regions determined lesser binding of RNA pol II to DNA fragments in the distal PAS region in non-malignant cells compared to malignant cells. Conversely, the opposite occurred in the proximal PAS region. Moreover, RNA immunoprecipitation (RIP) identified that U2 snRNP, a major component of RNA splicing complex that interacts with the 3′end of an intron, showed greater binding to the last intron of the FANCD2-V1 transcript in malignant cells compared to the non-malignant cells. Importantly, our data showed that in human tissue samples, the ratio of V2 /V1 expression in lung, bladder, or ovarian cancer correlates inversely with the tumor stages/grades. Therefore, these findings provide a previously unrecognized central player FANCD2-V2 and thus novel insights into human tumorigenesis, and indicate that V2/V1 can act as an effective biomarker in assisting the recognition of tumor malignance.

## INTRODUCTION

Fanconi Anemia (FA) is one of aging diseases, characterized with many developmental abnormalities and an extremely high incidence of both hematological and non-hematological malignancies [1–6]. At the cellular level, FA is characterized by chromosomal abnormalities and hypersensitivity to DNA crosslinking agents [1, 7, 8]. The similar sensitivity of FA cells from 21 groups (FANC-A, B, C, D1, D2, E, F, G, I, J, L, M, N, O, P, Q, R, S, T, U and V [2, 5, 6, 9–19]) to DNA crosslinking agents and the common clinical phenotype associated with each group suggest that the FA proteins all function in a common signaling transduction pathway (the FA pathway). The activation of this pathway occurs during DNA replication or upon DNA damage, especially when triggered by DNA crosslinking agents such as mitomycin C (MMC), diepoxybutane (DEB), and Cisplatin [20, 21]. Germline FA gene mutations have been directly associated with numerous cancers, such as breast, ovarian and pancreatic, owing to the defects related to FANCD1 /N /C, and/or /G [9, 22–25]. Specifically, mutations in FANCD1 (BRCA2) carry an 82% lifetime risk of breast cancer, and 23% risk of ovarian cancer [24, 25]. These genetic studies support the prediction made more than 40 years ago by Dr. Swift [26] that FA heterozygotes have an increased risk of cancer. However, the implications of the tumor suppressor function of the FA pathway just recently gained increasing interests in the field of cancer research.

Although FA genes do not share sequence similarity, their corresponding protein products are closely related [4]. Among 21 known FA family members, FANCD2 appears to be the focus of the FA pathway [9]. FANCD2 and its paralog FANCI are activated by the assembly of the FA proteins into a common nuclear protein complex, which acts as the E3 ubiquitin ligase to monoubiquitinate FANCD2. In response to DNA damage and during DNA replication, the activated /monoubiquitinated FANCD2 forms nuclear foci with other proteins (BRCA1, BRCA2, others) involved in DNA repair [11, 13, 22, 25, 27–30] to initiate nearly all currently known repair processes [4, 27, 31–35]. Taken together, the regulation of FANCD2 gene expression is essential for maintaining normal DNA repair and replication.

The functions of the FA pathway are heavily depending upon each FA gene encoded protein. How FA protein product is produced depends upon the multiple layers of regulatory processes, especially those involved in RNA processing [36, 37]. Poly(A) site choice has the potential to affect gene expression in many ways. The nature of the 3′-Untranslated Region of the mRNA (3′-UTR) is subject to change and affecting the stability and/or translatability of the final mRNA product [38]. By utilizing appropriate up- and downstream regulatory elements, polyadenylation can occur at certain positions within genes to effectively aggregate mRNA with an alternative open reading frame and/or a new 3′-UTR [39]. In addition, bioinformatics studies have suggested that 50% or more of all mammalian genes have more than one poly(A) site [40]. Presumably, this discovery reveals a possibility that alternative polyadenylation sites may regulate gene expression. This can be seen in the “switching” of immunoglobulin M heavy chain production from the membrane-bound to the secreted during B lymphocyte differentiation [41]. Alternative PAS is likely a widespread phenomenon, which contribute to fundamental gene transcriptional processes. In eukaryotes, alternative PAS not only generates functional proteomic diversity, but also plays an important role in regulating gene expressions [42]. However, the involvement of alternative PAS in FA gene expressions to affect the tumor suppressor functions of the FA pathway has never been reported.

Through sequence similarity searching from NCBI, we found a 60 bp longer version of FANCD2 coding cDNA, encoding a protein carrying more than 95% of homology with the known “FANCD2” protein. We named the newly recognized version of FANCD2, −“FANCD2-V2”−(V2), and −“FANCD2-V1”− (V1) for the long- known form. As this distinction has not been previously described, many of the reported functions of FANCD2 could reflect the properties of either V1, V2, or both. In this study, we are among the first to report an overlooked representative of the FA signaling pathway, which we termed FANCD2-V2. Our data show that this variant of FANCD2 exhibits greater association with non-malignant cells compared to malignant cells and properties of a potent tumor suppressor. Most importantly, we found that PAS plays a critical role in regulating the expression of different variants of FANCD2.

## RESULTS

### The overlooked FANCD2 variant (FANCD2-V2) appears to be a more potent tumor suppressor than the long-known one (FANCD2-V1)

**Figure 1:**
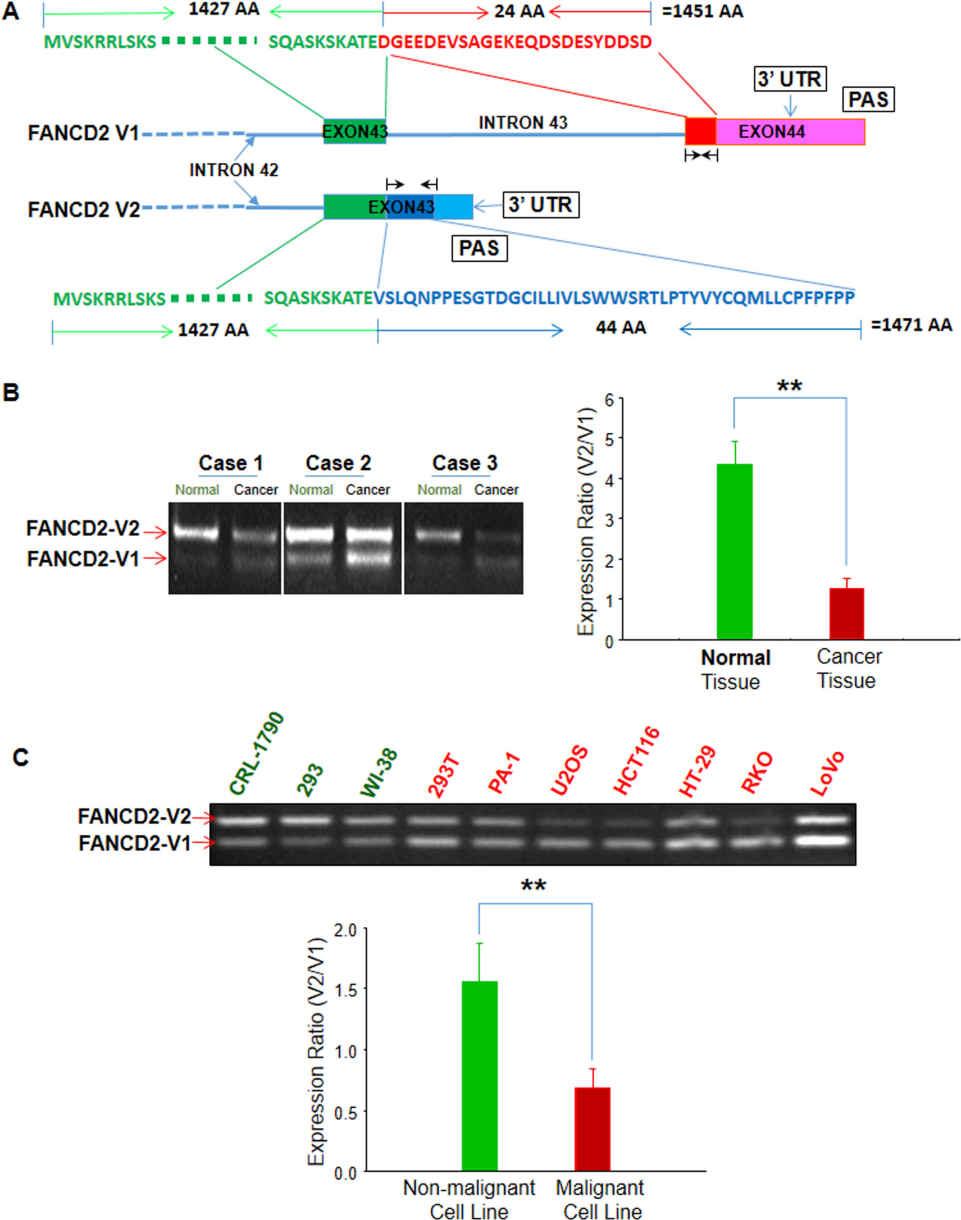
Two variants of FANCD2: namely “FANCD2-V1 (V1)” for the long-known FANCD2 and “FANCD2-V2 (V2)” for the overlooked one V2 is relatively highly expressed in benign cells or tissues as compared to V1. (**A**) FANCD2 gene contains two potential polyadenylation signaling motifs, which result in two proteinic variants: V1 and V2 that have a large common amino terminal (1427 AAs) (green-colored) and a 24 or 44 unique AA at the C-terminal respectively (red/V1 or blue/V2) (Black arrowheads indicate the RT-PCR primers used for detecting V1 or V2 mRNA expression.). (**B**) RT-PCR shows V1 or V2 relative expressions in malignant or matched non-malignant lung tissues. V2 is relatively high expressed in non-malignant tissues as compared to V1 (*t*-test, **p < 0.01). (**C**) RT-PCR shows V1 or V2 relative expressions in malignant or non-malignant human cell lines. V1 expression is relatively higher than V2 in malignant cells or transformed cells (PA-1, U2OS, HCT116, RKO, HT-29, LoVo and 293T) compared to non-malignant cells tested (CRL-1790, 293 and WI38) (*t*-test, **: *p* < 0.01). (All bar graphs were plotted from ImageJ quantification of RT-PCR results.)

Considering the importance of FANCD2 in the FA pathway, we investigated its potential homologs and found that, besides the long-known form of FANCD2 (NCBI RefSeq accession# NM_001018115.2 and NM_001319984.1, which code the same FANCD2 protein), there is an overlooked alternative variant (NCBI RefSeq accession# NM_033084.4). This newly discovered variant, which we named FANCD2-V2, has not been previously characterized. Henceforth, in order to both recognize and distinguish the two variants we have named the long-known form of FANCD2, FANCD2-V1. As shown in Figure 1A, the cDNA of V1 encodes 1451 amino acids (AAs), compared to V2, which encodes 1471 AAs. Additionally, both these variants share a large common region of 1427 AAs at the amino terminal. Next, we conducted RT-PCR to determine the V2-expression, using two sets of primers designed to detect specific regions of V2 and V1 respectively. In three sets of matched lung tissue samples (malignant versus adjacent non-malignant), we found that V2 is relatively, highly expressed compared to V1 in non-malignant tissues (Figure 1B), noting that the ratio of V2 over V1 (V2/V1) is higher in matched normal lung tissue samples compared to corresponding malignant tissue samples (Figure 1B). Similar results were also obtained in cell lines tested (Figure 1C), which suggest that V2 may possess more potent tumor suppressor capabilities than V1.

### RNA polymerase II is much less interactive with the distal-PAS region of FANCD2 gene in non-cancer cells compared to the cancer cells, and the usage of the distal-PAS defines V1 pre-mRNA

To understand how FANCD2-V2 expression is regulated, we thoroughly analyzed the gene structure of FANCD2. Inspection of the FANCD2 gene showed two potential polyadenylation sites, a proximal or distal site, associated with V2 and V1 respectively (Figure 1A) [42]. Accordingly, the synthesis of pre-mRNA by RNA Pol II is terminated earlier for V2 transcript compared to V1, attributed to the alternative polyadenylation sites we have identified (Figure 1A). Despite this, the corresponding transcript for V2 generates a longer version of mRNA, compared to V1. This is because V1 has an intact last intron, carrying both 5′ donor and 3′ acceptor sequences, which is missing in the V2 transcript, and the resulting partial intron completely was processed into the last exon of V2 mRNA. Underlying this, the two FANCD2 variants appear to depend on the location of either the proximal or distal PAS. Thus, we hypothesized that RNA Polymerase II activity may cease at the proximal PAS region in order to generate V2 pre-mRNA, whereas the distal PAS is employed for the production of the long-known V1 transcript [43, 44].

**Figure 2:**
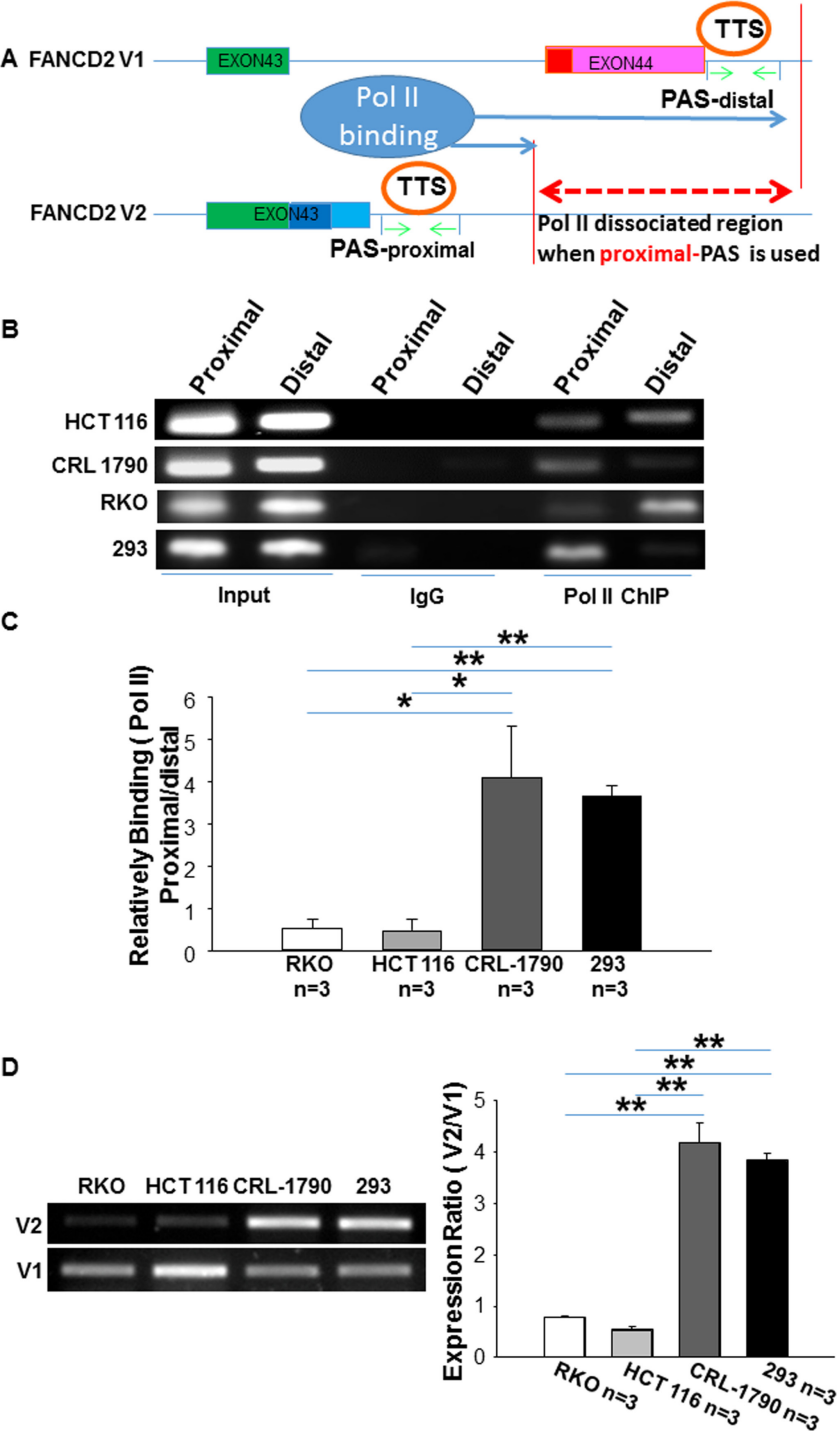
RNA polymerase II binds relatively more to the distal PAS region of FANCD2 gene in cancer cells as compared to the non-cancer cells (**A**) Alternative Transcription Termination Site (TTS) follows alternative PAS, and RNA pol II dissociates from DNA template at TTS and stops transcription [43], upon which DNA fragments after TTS can be distinguished by the binding capacity of pol II antibodies. As green arrowheads indicated, proximal or distal primer-bracketed regions pulled down by pol II antibodies are able to define the frequency of usage for proximal or distal PAS during the transcription. Therefore, the dash red line indicates the region where pol II is unable to binds when proximal-PAS is used. (**B**, **C**) Pol II antibodies pulled down relatively more DNA fragments located at the distal-PAS region in malignant cells (HCT116 or RKO) as compared to the non-malignant cells (CRL1790 or HEK293). *t*-test, *p < 0.05; **p < 0.01. (The bar-graph was plotted from Real-time qPCR.). (**D**) The V1 or V2 mRNA expression in these cells used for ChIP or RIP (next figure): V1 expression is relatively higher in non-malignant cells compared to malignant cells.

To test this, we performed RNA Pol II ChIP with two sets of ChIP PCR primers as illustrated in Figure 2A. Our data showed that DNA fragments at the distal PAS region pulled down by pol II antibodies was higher in malignant cells (RKO & HCT116) (Figure 2B, 2C), compared to non-malignant cells (CRL-1790 & HEK293). Accordingly, the binding between the DNA fragments at the distal PAS region and RNA pol II was reduced, and the low or high V2/V1 expression ratio was found respectively in these malignant or non-malignant cells (Figure 1C and Figure 2D). These results demonstrate that the alternative PAS plays an essential role in regulating the expression of V1 and V2, adding an additional level of complicity to the tumor suppressor roles of FA signaling.

### The interaction between U2 snRNPs and the last intron of V1 occurs more often in the same tested malignant cells as compared to the non-malignant cells

**Figure 3:**
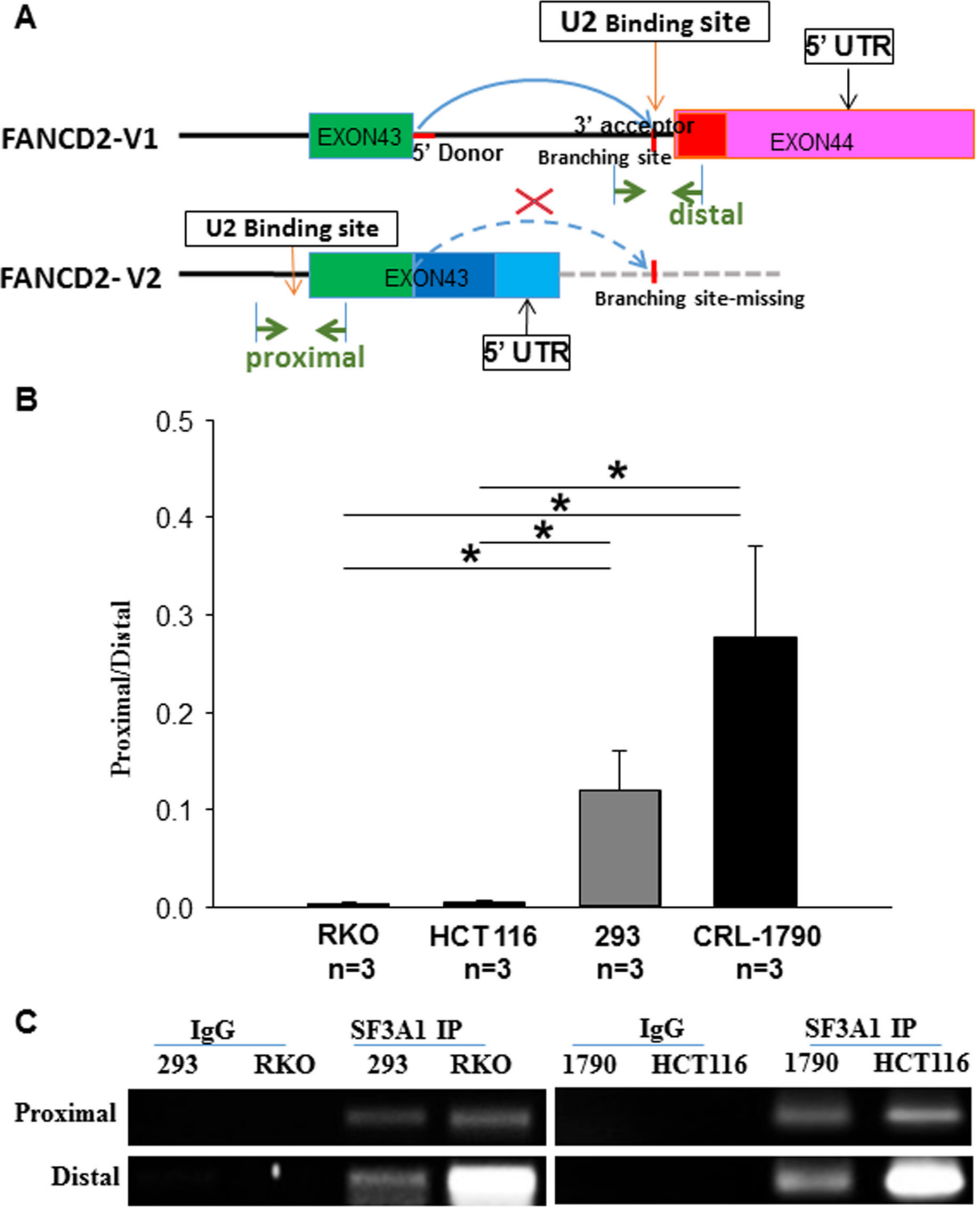
SF3A1 (the core component of U2 snRNP) RNA immunoprecipitation (RIP) shows that U2 snRNP interacts more with the last intron of V1 transcript in cancer cells as compared to the non-cancer cells (**A**) Schematic representation of the pre-mRNA splicing outline for V1 and V2 variants: when the proximal PAS is used, the resulting V2 transcripts lacks the last intron (the curve dash blue line), shown in the V1 transcript. The RIP-PCR primers were designed upon the regions marked with green arrowheads (distal or proximal primers were called accordingly for detecting relevant RNA fragments). RT-qPCR was used to detect RNA fragments pulled down by antibodies targeting SF3A1 (a key component of U2 snRNP). (**B**) The RT-qPCR product ratio, reflecting the relatively binding capacity between SF3A1 and the last intron of V1 or V2: U2 snRNPs interact relatively more with the last intron of V1-transcript in cancer cells as compared to the non-cancer cells. *t*-test, *p < 0.05; **p < 0.01. (**C**) Agarose gel images of resolved RT-qPCR products: those images show a good quality of RIP and validate the size of anticipated PCR products.

As illustrated in Figure 3A, the last intron in the primary V1 RNA transcript, stopping at the distal PAS region, contains a set of 5′ donor and 3′ acceptor sequences (red color); while the V2 transcript ends at the proximal PAS region only carries the 5′ donor site in the same part of pre-mRNA. Based upon the identification of two FANCD2 pre-mRNA transcripts (Figure 1A), we designed primers bracketing the 3′ donor site specific to the last intron in the V1 pre-mRNA. Concurrently, as the 3′ donor site of the last intron in the V2 pre-mRNA is present in both variants, we also designed another set of primers to act as a control. These two sets of primers (as indicated in the Figure 3A, proximal or distal) were used to conduct U2 snRNP RNA immunoprecipitation (RIP) (see green arrows). Again, using the same sets of cells for performing RNA pol II ChIP (Figure 2), we used the antibodies against SF3A1, the core component of U2 snRNP, to perform RNA immunoprecipitation followed by RT-qPCR-amplifying these two RNA fragments. Based upon observations, using the SF3A1 antibody to pull-down fragments representing the last V1 intron, binding was greater in malignant cells compared to non-malignant cells. This suggests that in malignant cells there is higher prevalence of the V1 primary transcripts compared to V2. This finding is consistent with the expression levels of both the V1 and V2 transcripts detected in the same cell lines (Figure 1C and Figure 2D). So far, we have provided evidence at both the DNA and RNA levels that supports our theory that the alternative PAS participates in the regulation of FANCD2-V1/V2 expression in a cell context dependent manner.

### FANCD2-V2 expression is present more in non-malignant tissues than in malignant ones

**Figure 4:**
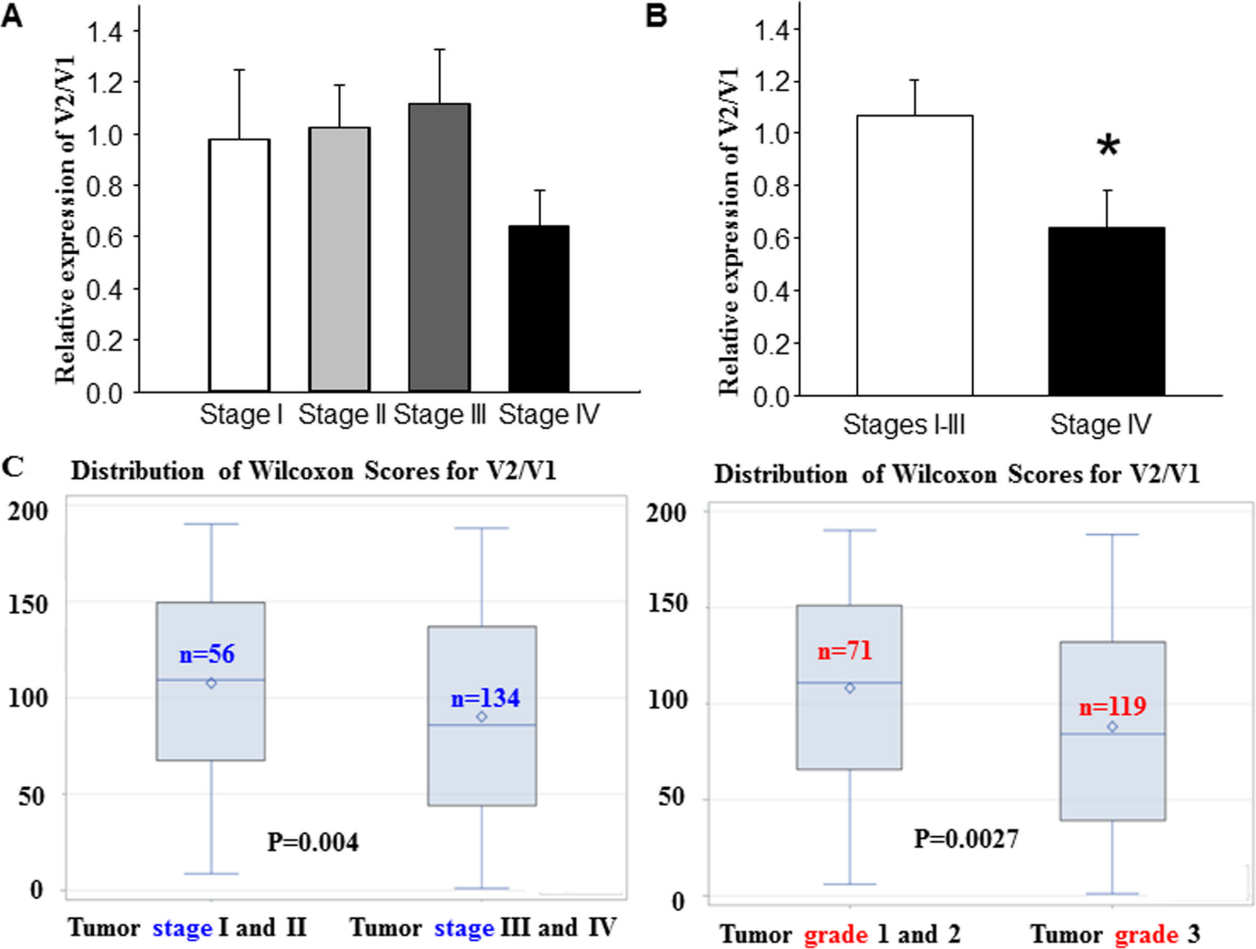
The ratio of FANCD2-V2 / FANCD2-V1 (mRNA expression) is inversely associated with human tumor stage or grade (**A**) By analyzing the specific exon junction sequence for V1 or V2 from the RNA sequence data set generated from human bladder cancer tissue samples (http://firebrowse.org/), we found the ratio of V2/V1 expression in stage IV tumors was lower than those in lower stag tumors. (**B**) The V2/V1 ratio in stage IV tumors is significantly lower than that in the combined stage I, II and III tumors. *t*-test, *p < 0.05. (**C**) The V2/V1 repression ratio is inversely associated with ovarian tumor stages (*r* = −0.206, *p* = 0.004) and grades (*r* = −0.226, *p* = 0.0027). Distribution of Wilcoxon scores was based upon two groups of 56 and 134 samples for tumor stage I&II, versus III&IV, respectively, to indicate the correlation between tumor stages and V2/V1 (left panel). Whereas two groups of 71 and 119 samples for tumor grade 1&2, versus 3 respectively were used for the score distribution to show the association between tumor grade and V2/V1 (right panel).

To establish the importance of this previously uncharacterized variant of FANCD2, V2, we aimed to validate its role as a tumor suppressor. To achieve this, we correlated its expression with the states of tested cells and tissues (non-malignant or malignant) shown in Figure 1B, 1C. Using publically available data sets, we analyzed specific sequence junctions of V1 and V2 (Figure 4A) in 402 human bladder cancer samples (The raw data were downloaded from http://firebrowse.org/). The ratio of V2/V1 expression was higher in stage I, II and III tumors compared to stage IV tumors. Additionally, the V2/V1 ratio in stage IV tumors were significantly lower than that in lower-stage tumors, even when combined (Figure 4B). This suggests a lower level of V2 expression may contribute to the tumor malignancy. To further validate our findings, we conducted real-time qPCR analyses on 200 cDNA samples derived from human ovarian cancer [45–47]. We found that the relative expression of V2 and V1 was largely associated with the tumor stages or grades (Figure 4C). In lower stage tumors, the relative expression of V2 is significantly higher than V1, whereas in higher stage tumors this ratio was much lower. Therefore, due to the identification of FANCD2-V2 our data indicate that V2 emerges to be a more potent tumor suppressor than V1. Furthermore, the V2/V1 ratio may serve as an effective biomarker in assisting the recognition of tumor malignancy.

## DISCUSSION

Since 1971, FA signaling has been argued to be responsible for various tumor suppressor roles by Dr. Swift [26]. However, in patients without FA, these roles were only empirically demonstrated since 2010 by the work we conducted [48–52]. With the hidden identity of a more potent central player, the relatively shorter form of FANCD2, V1, has been, unfortunately, recognized as the only representative of the FA pathway for decades. Therefore much of this pathway still remains elusive. Based on our present study, the prominent long-known form of FANCD2 (V1) may in fact signify abnormality, in the context of tumor suppression, and has mistakenly been considered normal. Our results indicate that V1 exhibits greater association with malignant cells, compared to non-malignant cells, and that V2 exhibits greater relative expression in normal cells, compared to malignant (Figure 1B, 1C and Figure 4). Therefore, we emphasize the importance of the V2/V1 ratio to more accurately describe the relationship between FANCD2 with cancer and aging. Surprisingly, this new revelation may indicate that the long-known form of FANCD2, V1, might be somewhat “oncogenic” owing to its relatively high expression levels in malignant cells/tissues, in conjunction with inefficient tumor suppressive activity compared to V2. This may provide a plausible explanation for the significant elevation observed in V1 expression in transformed cells. Thus, we question the validity of FANCD2-V1 as the orchestrator responsible for previously reports of FA signaling, given the possibility of an unnoticed system error.

In living cells, transcriptional variants, which differ in length and production are common, especially in human malignant cells. Importantly, intricate regulatory mechanisms underpin the diversity observed amongst these variants. At the DNA level, the local DNA structure, such as the B-B or A-A premeltons [53], a variety of DNA sequence islands including insulators [54] etc. Whereas, at the RNA level, exon skipping [55], lariat intron formation [56] and non-sense-mediated mRNA decay (NMD) [57] are just a few of the many examples, which affect the length and production of RNA variants. In this study, we highlight the alternative PAS as an important factor in a previously unrecognized mechanism contributing to the expression of two alternate forms of FANCD2. Based on the amazing similarity between qPCR statistics derived from RNA pol II ChIP and the expression levels of V1 and V2 (Figure 2C and 2D), we believe that the alternative PAS is a dominant cause of determining V1 or V2 expression in each given cell, and thus plays an essential role in the tumor suppressor function of FA signaling.

Compared to genetic, epigenetic, and other processes in RNA processing, PAS influences a fate of a gene function was under-reported. However, PAS is shown here to be an essential factor in determining the tumor suppressor function of the FA pathway, because the newly recognized version of FANCD2 (V2) appears to have tumor-suppressor function more potent than the version we have known for decades (V1) (Figure 1B, 1C and 4). Owing to the high homology between V1 and V2 (Figure 1A), the most known function of FANCD2, unfortunately, is a mixture of both depending on the ratio of their expression. The exact function of each form remains largely undefined, and the known functions of FA signaling are, thereby, not demonstrated as clearly as we thought before.

## MATERIALS AND METHODS

### Cell lines

All cell lines were obtained from the American Type Culture Collection (ATCC).

### RT-PCR and RT-qPCR

Cellular RNA was extracted by TRIzol Reagent (Life technologies). Reverse Transcription was performed using the Bio-Rad iScript Reverse Transcription kit as per the manufacturer's instruction.

Ovarian cancer tissue cDNA samples were provided by Dr. Herbert Yu [46, 47]. Real-time qPCR reactions were carried out with Power SYBR Green Master Mix (Thermo Fisher Scientific) by Applied Biosystems 7900HT Fast Real-Time PCR.

The following PCR primer sequences were used: FANCD2 V1 F, GAT GGT GAA GAA GAC GA and FANCD2 V1 R, GGT CTA ATC AGA GTC ATC A; FANCD2 V2 F, GTA TCT CTA CAA AAC CCA C and FANCD2 V2 R, GCT GTT ATG GAG GGA ATG.

### Chromatin immunoprecipitation (ChIP) assay

A ChIP assay was performed as previously described [58]. The primer sets were designed to encompass the region after the Transcription End Site (TES) and around the Transcription Termination Site (TTS; distal or proximal) of the FANCD2 gene. The sequences of used primers are as follows:

Distal forward primer: TGC CTC AGT TGC CTC ATT TAT, (reverse primer): GCA CCA GTC TCC ATA ATT TCT CT;

Proximal forward primer: GGC TCA CAC CTG TAA TCG TAG, (reverse primer): GTC TCG GGT TGG TCT TGA AA;

### RNA Immunoprecipitation (RIP) assay

Cells were harvested using trypsin and suspended into well isolated and subsequently crosslinked with 1% formaldehyde at room temperature for 10 min. After the reaction was quenched with 125 mM Glycine, the cells were washed 3 times in PBS and then resuspended in buffer A (5 mM PIPES pH 8.0, 85 mM KCl, 0.5% NP40, 1× complete protease inhibitor (Roche) and 50 μl/mL SUPERase In RNase inhibitor (Life technologies) and incubated on ice for 10 min. The pelleted crude nuclei fractions were centrifuged at 2500 g for 15 min at 4°C and then washed once in buffer A without NP40. The pellet was resuspended in RIP buffer (50 mM Tris-HCl pH 8.1, 150 mM NaCl, 0.1% SDS, 0.5% Deoxycholic Acid, 1% CHAPS, 5 mM EDTA, 1× complete protease inhibitor, and 50 μl/mL SUPERase In RNase inhibitor), and the solution was incubated on ice for 10 min. The lysate was sonicated in a Bioruptor Plus (Diagenode, SA), two times for 10 min each at high power while cycling for 30 seconds on and 30 seconds off. Following sonication, the lysate was pelleted via centrifugation at 13,000 RPM for 10 min to remove insoluble debris. The supernatant was diluted in IP buffer (50 mM Tris-HCl pH 8.1, 50 mM NaCl, 0.1% CHAPS, 1.2 mM EDTA, 1x complete protease inhibitor, and 50 u/mL SUPERase In RNase inhibitor) at a 1:1 ratio. After the solution was IgG pre-cleaned, a 5% aliquot of each sample was preserved in 1 mL of TRIzol Reagent (Life technologies), and this aliquot was used as the input control. The rest of the lysate was divided into two equal parts to which 2 μg of the SF3A1 antibody (Novus Biologicals) or the control rabbit IgG (Sigma-Aldrich) was added. All tubes were rotated at 4°C overnight, and then 50 μl of rec-Protein A-Sepharose 4B (Life Technologies) beads were added to each eppendorf tube, and then rotated for an additional 2 h at 4°C. The beads were washed 3 times using wash buffer (2 mM Tris-HCl [pH 8.1], 500 mM NaCl, 0.1% SDS, 1% CHAPS, and 50 u/mL SUPERase In RNase inhibitor), and then 1 mL of TRIzol Reagent was added to each tube. RNA was isolated from these samples, including the input controls, according to the manufacturer's instructions. RT-qPCR was performed using a SensiFAST SYBR Hi-ROX One-Step Kit (Bioline) in a StepOnePlus Real-Time PCR System (Life Technologies). The primer sequences that were used in these experiments are listed below.

Proximal (forward primer): TGT GTA TCC TCT AGG AGC TGT AT, (reverse primer): CTT CGT CTT CTT CAC CAT CCT AA;

Distal (forward primer): TGC CTG TAA ACT CAA CCT TCT C, (reverse primer): GGG ACT TAA TCT CTT CAC CCT AAA T.

### Statistics

Ovarian cancer RT-qPCR results were tested by Distribution of Wilcoxon Scores. The rest data were examined using two-tail student's *t*-test.
